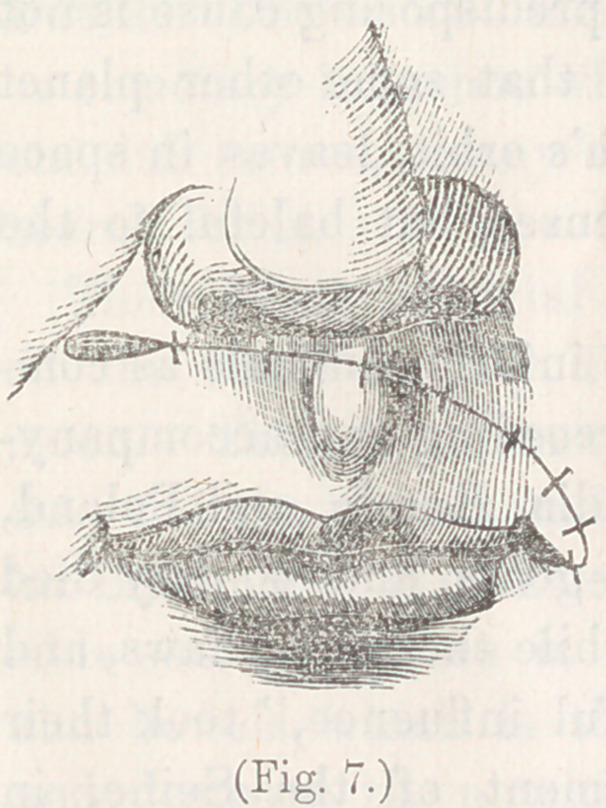# A New Plastic Operation for Certain Deformities of the Face

**Published:** 1866-08

**Authors:** E. Andrews

**Affiliations:** Professor of Surgery in Chicago Medical College


ARTICLE XXVI.

### A NEW PLASTIC OPERATION FOR CERTAIN DEFORMITIES OF THE FACE.

BY E. ANDREWS, M.D., Prof. of Surgery in Chicago Medical College.

It is very difficult to say that any plastic operation is absolutely new, because so many have been performed which have never been published, that it is probable that almost every possible principle has been made use of by some one. Still a careful examination of both European and American authorities have failed to furnish me any president for a mode of operating which I have used for some time, in certain cases, and which I deem very valuable. I, therefore, judge it to be a new operation. If, however, it shall appear that any one has practiced it before me, I will cheerfully resign my claim to priority, simply hoping that so useful a principle will not be allowed again to slumber where it cannot be found for practical purposes.

There are very serious objections to that whole class of plastic operations which consist in transplanting flaps of integument which have been completely dissected out of their beds, and only retain vascular connections at one extremity. They have so feeble a circulation that they run great risk of failure to adhere in their new locations, and may even mortify from slight causes. Again they are surrounded on all sides of their new location by a cicatrix which, after some months of contraction, very much restricts the flow of blood to them. The amount of obstruction offered to the passage of blood by an old cicatrix is very great. Hence such flaps, though they look extremely well at first, after a time begin to be atrophied, and often by the unexpected amount of their contraction, reproduce the deformity they at first corrected. I have seen old flaps of this sort which had shrunk to one-third of their original diameter.

Impressed by these difficulties, I have sought to devise methods which will allow of keeping all the more important arterial trunks untouched in the flap. My first attempt was in the case represented in Figs. 1 and 2. The deformity consisted in the loss of the left *ala* of the nose, nearly up to the nasal bones. Observing that the stump of the cartilage had a smooth, firm border, well healed, and exactly of the shape to make a good edge of the nostril, I determined to make use of it for that purpose. Making an incision, therefore, along the semicircular dotted line, I produced a flap broadest at the base, and receiving on its interior surface the abundant branches of the facial artery. Dissecting up from the nasal bones a few rather firm adhesions, I found that, owing to the looseness of the tissues of the cheek, it was easy to revolve the flap on its centre without cutting its subjacent connective tissue and

(Fig. 1.)

(Fig. 2.)

vessels. I, therefore, refreshed the edge of the nose in a strip marked by the dotted line from the tip of the flap to the tip of the nose; then revolving the flap on its centre, I placed its cicatrized border in the position of the natural edge of the nostril, and fastened the whole with sutures, as shown in fig. 2. The outer extremity of the incision folded its edges by each other in such a manner as to allow of complete closure of the wound. The result was most excellent.

From the experience of this instance, I am led to believe that often in case of the loss of the whole cartilaginous portion of the nose, the best method of restoration would be to bring in two flaps in the same manner, one from each side, instead of transplanting tissue from the forehead.

The second operation was an application of the same principle to the eyelid, for the cure of entropium. The lower lid of a patient had been drawn down and everted, by the contraction of a cicatrix following a burn, and presented the appearance shown in fig. 3.

(Fig. 3.)

On measurement, the tarsal border of the everted lid was found to be so stretched by its downward traction that it was about one-third longer than its fellow, requiring to be shortened by that amount. I commenced by making an incision from the inner canthus along the line of junction between the mucous membrane and the skin, separating the two membranes freely from each other, until the knife had traversed about one-third of the length of the tarsal border; then turning the edge downward, I made a semicircular sweep in the direction shown by the dotted line in fig. 3. A short incision was made from the outer extremity of this sweep, upward and inward, to liberate the flap from the traction of the skin of the temporal region. It will be seen at a glance, that this flap not only had a broad vascular communication through the conjunctiva and the skin near the external canthus, but what is still more important, it preserved intact the whole group of arterial twigs given off from the infra-orbital artery. By experiment, I found the subcutaneous tissue of the flap so loose that by simply dissecting up a little of its lower edge, it was easy to slide or revolve the whole of it upward and inward to its proper position against the eye.

(Fig. 4.)

It was revolved in this manner, therefore, until its apex, that is, the point where the incision turned downward from the tarsal border, was carried upward and inward quite into the inner canthus, where it was lodged and stitched into the incision between the mucous membrane and the integument. Figure 4, represents the flap in its place, with the sutures inserted, but not yet tied. The flap having being cut with the lower edge corresponding to the direction of its curve of revolution, filled without difficulty the place intended, leaving a vacant triangular space near the malar bone. The sutures sloped inward, as shown in the cut, in order to hold the flap well up to its place, and the triangular vacancy was closed by three stitches, drawing its sides to the centre, as shown in the figure. On tying the sutures, the whole presented the appearance displayed in fig. 5.

(Fig. 5.)

This patient had an attack of traumatic erysipelas, which destroyed a part of the adhesions; nevertheless, the result was very good, though not quite perfect. In another case of the same sort, I took the precaution to free the flap a little more from its subcutaneous adhesions, and to place it higher up against the eye, to allow for a little settling or depression, which occurs after the operation in consequence of the elasticity of the tissues. This gave a still better result. The punctum lachrymal in this case had been destroyed, so that no care was required to avoid it; but when it exists, it should be left on the conjunctival side of the incision, and care be taken not to injure the lachrymal duct. During the healing, and for some time after, the flap was kept supported by adhesive straps.

(Fig. 6.)

Encouraged by my experience in these cases, I have applied the same principle to the restoration of lost portions of the lips. I have operated on four such cases in this manner, with the most perfect success. Fig. 6, represents the first of them. The left half of the mouth had been reduced to the form of an open triangle by a previous attack of gangrene, which destroyed a portion of the cheek and about one-quarter of the upper-lip. After the parts had healed, they presented the appearance shown in this figure. The prolabium of the upper-lip was drawn up so as to constitute the inner side of the triangle; the old cicatrized edge of of the cheek constituted its outer side, and the prolabium of the lower-lip its base. Taking the scalpel, I pared the cicatrized edge of the cheek, and then cut through the whole thickness of the lip, along the curve shown by the dotted line. The flap was then easily revolved into the position

(Fig. 7.)

shown in fig. 7, and fastened with sutures and adhesive straps. The lip is always favorably disposed for adhesive union, and the results in all my cases on this organ were all that could be desired.

This method of operating is, of course, not adapted to all facial deformities; but where it is applicable, it affords the following important advantages:—

1st. The flap is better nourished, and, therefore, less liable to mortify, or fail of adhesion, than in any operation hitherto devised.

2d. For the same reason, it is less liable to a slow subsequent atrophy.

3d. There is no open spot left after the operation to heal by granulation and form an unsightly scar.

## Figures and Tables

**Fig. 1. f1:**
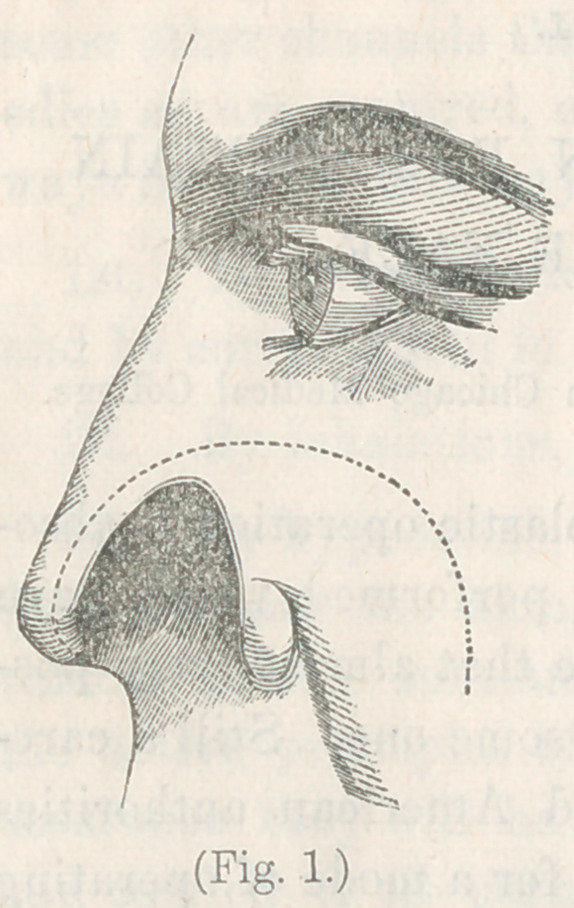


**Fig. 2. f2:**
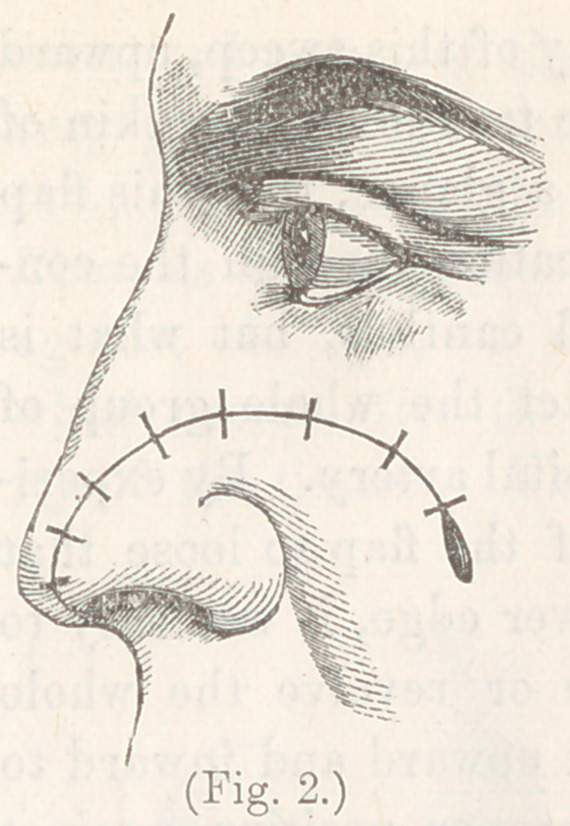


**Fig. 3. f3:**
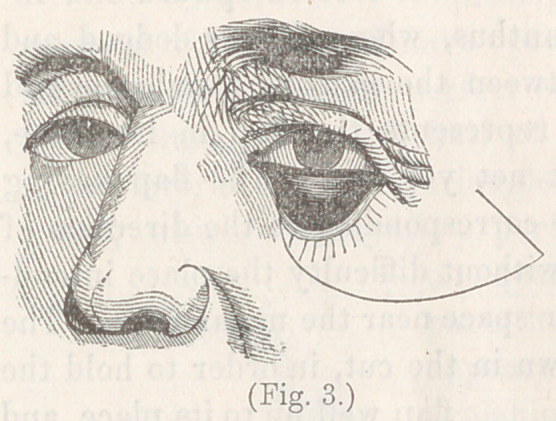


**Fig. 4. f4:**
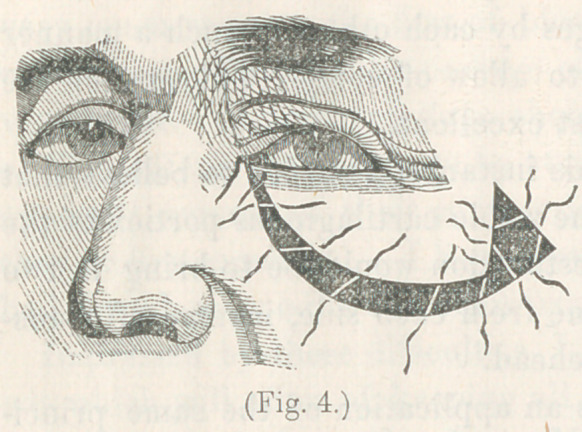


**Fig. 5. f5:**
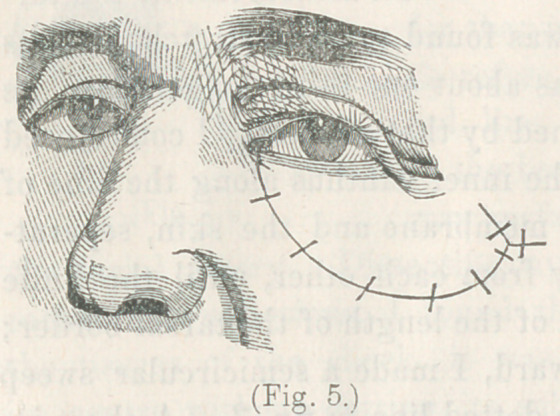


**Fig. 6. f6:**
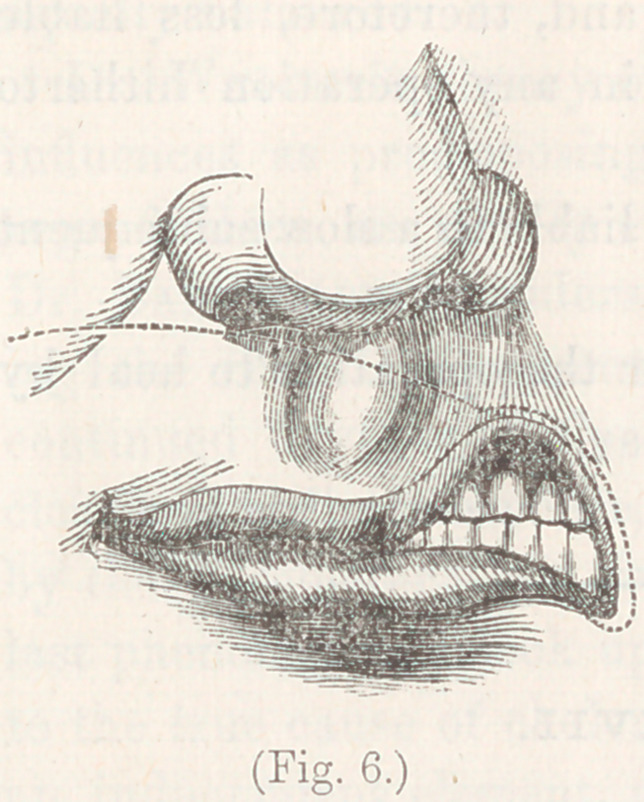


**Fig. 7. f7:**